# Biologikatherapie bei schwer erkrankten Patienten mit „NSAID-exacerbated respiratory disease“ und stattgehabter ASS‑Desaktivierung: Ergebnisse einer multizentrischen Studie

**DOI:** 10.1007/s00106-024-01433-y

**Published:** 2024-03-11

**Authors:** Anna-Rebekka Staufenberg, Hanna K. Frankenberger, Ulrike Förster-Ruhrmann, Franziska C. Spahn, Ludger Klimek, Kai Fruth, Clemens Stihl, Christoph Matthias, Moritz Gröger, Jan Hagemann

**Affiliations:** 1 Hals‑, Nasen‑, Ohrenklinik und Poliklinik, Universitätsmedizin Mainz, Langenbeckstr. 1, 55131 Mainz, Deutschland; 2 Klinik und Poliklinik für Hals-Nasen-Ohrenheilkunde, Klinikum der Ludwig-Maximilians-Universität München, Campus Großhadern, München, Deutschland; 3 Klinik für Hals‑, Nasen‑, Ohrenheilkunde, Charité – Universitätsmedizin Berlin, Berlin, Deutschland; 4 Zentrum für Rhinologie und Allergologie, An den Quellen 10, Wiesbaden, Deutschland; 5 HNO Zentrum Mainz, Emmeransstr. 9, 55161 Mainz, Deutschland

**Keywords:** Atemwegsentzündung, Acetylsalicylsäureinduziertes Asthma, Hypersensitivität, Rhinosinusitis, Klinische Real-World-Studien, Airway inflammation, Aspirin-induced asthma, Rhinosinusitis, Hypersensitivity, Real-world clinical trials

## Abstract

**Hintergrund:**

Die chronische Rhinosinusitis mit Nasenpolypen (CRSwNP) ist eine chronisch-entzündliche Erkrankung der oberen Atemwege mit starker Beeinträchtigung der Lebensqualität. Die von „NSAID-exacerbated respiratory disease“ (NERD) betroffenen Patienten weisen i. d. R. ein hochdynamisches Wiederauftreten der Beschwerden nach Operation, oraler Kortikosteroidgabe und Acetylsalicylsäuredesensibilisierung (ATAD) auf. Die Add-on-Biologikatherapie hat die Wahl des therapeutischen Konzepts grundlegend verändert, Subgruppen wie der der NERD sind jedoch unzureichend untersucht. Ziel der vorliegenden Arbeit ist, es eine multizentrische retrospektive Studie über die Add-on-Therapie mit Dupilumab, Omalizumab und Mepolizumab bei Patienten mit gesichertem NERD vorzustellen.

**Methode:**

Es handelt sich um eine retrospektive Kohortenstudie von Patienten (NERD+, Status nach ATAD) dreier Referenzzentren in Deutschland (München, Mainz, Berlin). Subjektive und objektive Parameter wurden nach 4/8/12 Monaten in Übereinstimmung mit EPOS/EUFOREA-Richtlinien (European Position Paper on Rhinosinusitis and Nasal Polyps/European Forum for Research and Education in Allergy and Airway Diseases) erhoben. Die Auswahl der Biologika erfolgte je nach Verfügbarkeit und Patientencharakteristik.

**Ergebnisse:**

Behandlungen wurden bei *n* = 122 Patienten mit CRSwNP und NERD begonnen. Der endoskopische Polypenscore, der SNOT-22-Fragebogen-Score (Sino-Nasal Outcome Test), der visuelle Analogskala-Score für die Gesamtsymptome/Schwere der Erkrankung und der Geruchssinn (psychophysische Tests mit Sniffin’Sticks/Brief Smell Identification Test, B‑SIT, Fa. Sensonics, Inc., Haddon Heights, NJ, USA) verbesserten sich signifikant nach 4 bzw. 12 Monaten Zusatztherapie (*p* < 0,0001). Alle 3 Biologika führten zu einer signifikanten Verbesserung eines oder mehrerer Krankheitsparameter. Unerwünschte Ereignisse waren nicht lebensbedrohlich, führten aber in 4 Fällen zu einem Wechsel des Biologikums. Die Patienten bewerteten die Biologikatherapie signifikant besser als ATAD, mit einer besseren langfristigen Kontrolle der Krankheit.

**Schlussfolgerung:**

Die Add-on Biologikatherapie ist wirksam, sicher und wird in der Gruppe der CRSwNP + NERD-Patienten weitgehend akzeptiert. Künftige Studien könnten personalisierte Algorithmen mit sequenzieller Chirurgie, ATAD und/oder Biologikatherapie ermöglichen.

## Abstract (en)

**Background:**

Chronic rhinosinusitis with nasal polyps (CRSwNP) is a type‑2 inflammatory disease of the upper airways, with severe impairment of quality of life. Persons affected by NSAID-exacerbated respiratory disease (NERD) usually present with highly dynamic recurrence of polyps and disease despite prior treatment with sinus surgeries, oral corticosteroids, and aspirin desensitization (ATAD). Biologic therapy has fundamentally changed the choice of therapeutic concept; however, limited data exist on subgroups such as NERD patients. The aim of the current article is to report on a multicenter retrospective study on add-on therapy with dupilumab, omalizumab, and mepolizumab in patients with NERD.

**Methods:**

This is a retrospective cohort study of patients (NERD+, status after ATAD) in three reference centers in Germany (Munich, Mainz, Berlin). Subjective and objective parameters were collected at 4, 8, and 12 months after biologic therapy initiation in accordance with current EPOS/EUFOREA (European Position Paper on Rhinosinusitis and Nasal Polyps/European Forum for Research and Education in Allergy and Airway Diseases) guidelines. Biologic agents were chosen depending on availability and patient characteristics.

**Results:**

Treatment was commenced in 122 patients meeting the criteria for CRSwNP and NERD. The endoscopic polyp score, SNOT-22 questionnaire score, visual analogue scoring of total symptoms/severity of disease, and sense of smell (psychophysical testing with Sniffin’Sticks/Brief Smell Identification Test, B‑SIT; Sensonics, Inc., Haddon Heights, NJ, USA) improved significantly after 4 and 12 months of add-on therapy (*p* < 0.0001). All three biologic agents significantly improved one or more disease parameter. Adverse events were not life threatening but led to change of biologic agent in 4 cases. Patients rated biologic therapy significantly better than ATAD, with improved long-term disease control.

**Conclusion:**

Add-on biologic therapy is effective, safe, and widely accepted among CRSwNP + NERD patients. Future studies might allow for personalized algorithms with sequential surgery, ATAD, and/or biologic therapy.

## Einleitung

Die chronische Rhinosinusitis (CRS) weist in Europa eine Prävalenz von 11 % auf [1]. Die CRS umfasst die vornehmlichen Phänotypen CRS ohne Polyposis nasi (CRSsNP) sowie mit Polyposis nasi (CRSwNP) [2]. Letztere ist eine chronische, schwer zu kontrollierende Erkrankung der oberen Atemwege, und viele der betroffenen Patienten sind stark in ihrer Lebensqualität eingeschränkt [3]. Die Erkrankung führt zu vielfältigen Symptomen des gesamten Respirationstrakts, am häufigsten jedoch zu nasalem Ausfluss, nasaler Obstruktion, Druckgefühl oder Gesichtsschmerzen und Geruchssinnverlust. Das oberste Therapieziel lautet daher, eine gute Symptomkontrolle zu erreichen. Die Standardtherapie bestand im Kern lange Zeit aus der topischen und oralen Gabe von Kortikosteroiden sowie der Nasennebenhöhlenoperation [2]. Seit wenigen Jahren steht auch die gezielte Zusatztherapie mit Biologika zur Verfügung, um nicht ausreichend kontrollierte Patienten zu behandeln [4]. Seit 2022 stellt diese per Beschluss des gemeinsamen Bundesausschusses (G-BA) die Standardtherapie für diese Patientengruppe dar, was zu mehr Verordnungssicherheit innerhalb der gegebenen Rahmenbedingungen geführt hat.

Eine besondere Patientengruppe innerhalb der CRSwNP bildet in 5–10 % der CRS-Fälle die Gruppe derjenigen, die von der „NSAID-exacerbated respiratory disease“ (NERD; Synonyme: AERD, M. Widal, M. Samter, Samter-Trias, Salicylatunverträglichkeit) betroffen sind [5]. Dies bezeichnet das gleichzeitige Vorliegen der CRSwNP, einer Nichtsteroidale-Analgetika(NSAID)-Intoleranz und eines Asthma bronchiale [9, 10]. Typischerweise ist der Endotyp bei NERD stark Interleukin(IL)-5-positiv mit schwerer Eosinophilie und erhöhten Spiegeln von IL‑4, IL-13, Eotaxin und eosinophiles kationisches Protein (ECP) [6]. Bei den Betroffenen liegt zudem nachweislich ein Ungleichgewicht der aus Arachidonsäure gebildeten Eicosanoide (Cysteinyl-Leukotrien E4/Cys-LTE4) und den Prostaglandinen (PGD2 und PGE2) vor. Dies führt zu einer verminderten PGE2-Synthese und einer Überproduktion von Cys-LT [7]. Von Szczeklik et al. wurde 1975 erstmals der nichtallergische Pathomechanismus respiratorischer Reaktionen nach Einnahme von NSAID durch den Nachweis der Inhibition der Prostaglandin(PG)-Synthese beschrieben [8]. Die Patientengruppe mit NERD ist auch deswegen besonders, da ein hohes Risiko für Rezidivpolypenwachstum mit entsprechend stärker ausgeprägten Symptomen, im Vergleich zu Nicht-NERD-Patienten, vorliegt. Die Diagnosestellung eines NERD kann anamnestisch, oder durch Provokationstestung mittels ASS-Gabe (p.o., intranasal, i.v.) erfolgen [9].

Neben wenig vielversprechenden Ansätzen einer salicylatarmen Diät und einer in Deutschland hierfür nicht zugelassenen Therapie mit Leukotrienantagonisten steht als besondere NERD-Therapie in erster Linie die sog. adaptive Desaktivierung mittels niedrigdosierter Dauergabe von Acetylsalicylsäure ASS zur Verfügung („aspirin therapy after desensitization“, ATAD). Sie basiert auf der sog. Refraktärphase nach Einnahme von ASS und wird i. d. R. kurz nach einer erfolgten Nebenhöhlenoperation begonnen, um die Intervalle zwischen chirurgischen Nebenhöhleneingriffen zu verlängern und die Krankheit zu kontrollieren. Mehrere Studien konnten den Nutzen der ATAD durch verbesserte sinunasale Symptome, Lebensqualität und Riechvermögen zeigen [10–12]. Die Wirksamkeit der ATAD in der Regelversorgung hinkt den Studienergebnissen oft nach, da die Therapieadhärenz der Dauereinnahme durch beispielsweise gastrale Beschwerden, Blutungskomplikationen und fehlenden Therapieerfolg stark reduziert ist [13]. Direkte Head-to-Head-Vergleiche bezüglich der Wirksamkeit zwischen den international sich unterscheidenden Dosierungen von ASS (zwischen 100–1300 mg p.o./Tag) gibt es nicht, und eine Fehldosierung bei bestimmten Patienten scheint zumindest denkbar [14].

Biologika stellen neuere Therapiealternativen für die Behandlung der schweren, unkontrollierten CRSwNP mit NERD dar. Die Ergebnisse der Phase-III-Zulassungsstudien ergaben eine hochsignifikante Reduktion sinunasaler Beschwerden und der nasalen Polyposis, was bislang zur Zulassung von Dupilumab, Omalizumab und Mepolizumab als Zusatztherapie („Add-on“) zur Basistherapie mit nasalen Steroiden führte [15–17]. Omalizumab wird alle 2–4 Wochen verabreicht und hindert freies IgE an der Bindung an Mastzellen, Basophilen und Eosinophilen [18]. In 4‑wöchentlichen Intervallen zu applizierendes Mepolizumab richtet sich gegen IL‑5 und hemmt die Proliferation, Migration und Aktivierung von eosinophilen Granulozyten. Dupilumab ist alle 2 Wochen zu verabreichen und richtet sich gegen die Alpha-Untereinheit des IL-4-Rezeptors und somit IL‑4 und -13-vermittelte Signalwege. Die Wirksamkeit der Biologikatherapie für die Patientengruppe mit NERD konnte bereits anhand von Post-hoc-Analysen der Zulassungsstudien der jeweiligen Präparate sowie anhand weniger klinischer Beobachtungen und kleinerer Studien vermutet werden [19–26]. Bislang existieren keine ausreichenden Ergebnisse über Sicherheit, Therapieansprechen und Therapieakzeptanz verschiedener Biologika in der Gruppe der NERD-Patienten. Die Autoren berichten über multizentrische, retrospektive Real-World-Daten zum Einsatz von Biologika bei CRSwNP mit stattgehabter ATAD bei ärztlich diagnostiziertem NERD und der Entwicklung zu festgelegten Zeitpunkten 4 und 12 Monaten nach Therapiebeginn.

## Methoden

Es handelt sich um eine retrospektive Studie an 114 Patienten mit NERD, die im Zeitraum von 2019 bis 2023 in prospektiven Kohorten entweder an der HNO-Klinik der Universitätsmedizin Mainz, der HNO-Klinik Charité – Universitätsmedizin Berlin oder der HNO-Klinik des Klinikums der Universität München (LMU) eine Add-on-Behandlung mit Biologika begannen und von denen Daten zu mindestens einer Verlaufskontrolle nach 4/8/12 Monaten Therapie erhoben werden konnten. Die jeweiligen Ethikkommissionen der Länder hatten ihr zustimmendes Votum gegeben, und die Patienten stimmten einer anonymisierten Verwendung ihrer Daten zu. Das Vorliegen eines NERD wurde gemäß den EPOS-Empfehlungen (European Position Paper on Rhinosinusitis and Nasal Polyps) entweder anamnestisch oder durch orale Provokationstestung geprüft und ärztlich bestätigt. Die Therapieindikation für die Biologikatherapie erfolgte auf Basis der jeweils als aktuell anzusehenden wissenschaftlichen Empfehlungen sowie konform mit der Fachinformation („in-label“) [4, 27–30] und nach einer Aufklärung der Patienten über Therapiealternativen. Die Auswahl des Präparats war für einen Großteil des Studienzeitraums (bis Frühjahr 2021) durch die erst später erteilte Zulassung von Omalizumab und Mepolizumab limitiert. Kamen danach mehrere Präparate gemäß Fachinformation und Leitlinien/Positionspapiere infrage, wurde gemeinsam mit den Patienten in einem Beratungsgespräch eine Präparatewahl vorgenommen. Die Basistherapie mit topischen Steroiden wurde fortgesetzt. Vor Beginn der Therapie wurden subjektive und objektive Befunde und Biomarker in Bezug auf die Atemwegserkrankung erhoben: Visuelle Analogskalen zu Krankheitsaktivität und Riechvermögen, endoskopischer Polypenscore [31], psychophysikalische Riechprüfung (Sniffin’Sticks-16-Item-Identifikationstest, 12-Item-Screening Test, 12-Item-B-SIT), SNOT-22-Fragebogen (Sino-Nasal Outcome Test) [32], eosinophile Granulozyten Zellen im Blut, IgE im Serum, Eosinophileninfiltrat in der Schleimhaut (ohne zusätzliche Probenentnahme), Allergiediagnostik (SX1, SPT, RAST). Zur Vergleichbarkeit der verschiedenen Riechprüfungen wurden Quotienten und prozentuale Veränderungen herangezogen. Weitere Parameter vgl. Tab. 1. Die Untersuchungen wurden möglichst vollständig in regelmäßigen Abständen wiederholt. Nebenwirkungen wurden dokumentiert (Tab. 2). Die Evaluation der Therapiefortsetzung erfolgte gemäß aktuellen Empfehlungen [28]. Es wurden neubegonnene Biologikabehandlungen untersucht, sodass einige wenige Patienten nach Präparatewechsel in mehreren Präparategruppen erscheinen. Die Patienten wurden zudem telefonisch/per Post kontaktiert, um Fragen zur Therapiebewertung und -akzeptanz zu beantworten. Mittels GraphPad Prism 10 (GraphPad Software, 225 Franklin Street. Fl. 26, Boston, MA, USA) erfolgte die statistische und grafische Auswertung. Statistische Signifikanz wurde mit * markiert und durch Abstufungen wie folgt definiert: * = *p* < 0,05; ** = *p* < 0,01; *** = *p* < 0,001; **** = *p* < 0,0001.

**Tab. 1:** Patientenkollektiv

	Baseline	12 Monate Follow-up	*p*-Wert^b^
	*n* = 122	*n* = 92	
Geschlecht, weiblich (%)	49,1	–	–
NERD (%)	100	–	–
ATAD (ASS-Desaktivierung) in Vorgeschichte (%)	100	–	–
Alter (Jahre)	52,1 (26–77)	–	–
VAS Gesamt (0 < 10)	7,6 (7,1–8,0)	2,7 (2,2–3,2)	< 0,0001*
VAS Riechminderung (0 < 10)	8,7 (7,9–9,5)	4,6 (2,5–6,6)	< 0,0001*
SNOT-22	64,2 (60,8–67,7)	27,2 (22,7–31,7)	< 0,0001*
Endoskopischer Polypenscore	5,1 (4,8‑5,4)	1,1 (0,8–1,4)	< 0,0001*
Veränderung Riechvermögen/Baseline in % (Sniffin’Sticks/B-SIT)	–	28,73 (22,1–35,4)	< 0,0001*
Rhinomanometrie (ml/s) ohne Abschwellen	436,4 (312,8–560)	466,6 (320,8–612,4)	0,7661
Eosinophile Granulozyten (Zellen/µl)	352,9 (278–427,7)	607,4 (381,7–833,2)	0,0063*
Gesamt-IgE (U/ml)	263,6 (187,2–339,9)	91,2 (32,9–149,4)	0,0416*
Allergiescreening positiv (SX1; Anzahl *n*)	53 von 103	–	–
Gewebseosinophilie (0–3, Mittelwert)^a^ (*n* = 95)	2,3	–	–
Anzahl der NNH-Operationen (Mittelwert)	3,1	–	–
Krankheitskontrolle:			
SNOT-22 < 40 Punkten & VAS < 5 (Anzahl *n*)	4	51	< 0,0001*
SNOT-22 > 40 Punkten & VAS > 5 (Anzahl *n*)	76	9	
Begonnene Therapien (alphab. sortiert; Anzahl *n*)	122	92	–
Dupilumab	112	84	–
Mepolizumab	4	2	–
Omalizumab	6	6	–
Präparatewechsel (Anzahl *n*)	–	8	–
Begonnene Therapien (alphab. sortiert; Polypenscore)			
Dupilumab	5,1 (4,7–5,5)	1,1 (0,8–1,5)	< 0,0001*
Mepolizumab	5,2 (2,9–7,6)	2,0 (2,0–2,0)	0,0446*
Omalizumab	3,5 (1,3–5,7)	3,0 (8,8–5,2)	0,6867
Begonnene Therapien (alphab. sortiert; SNOT-22)			
Dupilumab	64,2 (60,5–67,7)	26,9 (21,9–31,9)	< 0,0001*
Mepolizumab	64,6 (42,3–86,8)	31,75 (−7,1–70,6)	0,0517
Omalizumab	51,86 (24,5–79,2)	36,0 (25,2–46,8)	0,3978
Begonnene Therapien (alphab. sortiert; Veränderung Riechvermögen/Baseline in %) ^c^			
Dupilumab	–	29,3 (22,1–36,4)	< 0,0001*
Mepolizumab	–	30,6 (16,6–42,5)	0,6917
Omalizumab	–	12,5 (−3–28)	0,5862
Begonnene Therapien (alphab. sortiert; VAS gesamt) ^c^			
Dupilumab	8,2 (7,8–8,6)	2,6 (2,1–3,1)	< 0,0001*
Mepolizumab	7,0 (−18,4–32,4)	4,2 (0,2–8,2)	0,2351
Omalizumab	7,3 (3,5–11,0)	3,3 (1,7–4,8)	0,0201*
Rescue-Therapien (orale Steroide, NNH-Op.; Anzahl* n)*	–	0	–

In Klammern: 95%-Konfidenzintervall, 95%-KII

*ATAD *Acetylsalicylsäuredesensibilisierung, *ASS *Acetylsalicylsäure,* B‑SIT *Brief Smell Identification Test (Fa. Sensonics, Haddon Heights, NJ, USA), *NERD *„NSAID-exacerbated respiratory disease“* NNH-Op.* Nasennebenhöhlen-Operation, *SNOT* Sino-Nasal Outcome Test,* VAS* visuelle Analogskala

^a^ 0 = keine, 1 = geringgradig, 2 = mittelgradig, 3 = hochgradig

^b^ „Turkey’s/Dunnett’s multiple comparison test“/t-Test/Fisher-Test

^c^ statistisch wurden die Absolutwerte von der Baseline und vom 12-Monats-Follow-up betrachtet

**Tab. 2:** Unerwünschte Nebenwirkungen

Nebenwirkungen/„adverse events“ (*n* = 22)	Anzahl *n*	Präparat
Rötung/Urtikaria Einstichregion	7	Dupilumab
Kopfschmerzen	4	Dupilumab
Fatigue und Gliederschmerzen/Arthralgien	4	Dupilumab
Schwindel	1	Dupilumab
Augenbrennen/Augenjucken	1	Dupilumab
Epistaxis	1	Omalizumab
Alopezie der Beine	1	Dupilumab
Gewichtszunahme	1	Dupilumab
Eosinophilie, die zum Absetzen des Präparats führt	1	Dupilumab
Rhagaden an Akren	1	Dupilumab
Therapiewechsel aufgrund von Nebenwirkungen (Eosinophilie, Urtikaria, Rhagaden, Gewichtszunahme)	4	Dupilumab

## Ergebnisse

Es wurden 122 Biologikatherapien bei 114 NERD-Patienten begonnen, von denen 92 Therapien das Follow-up zum Zeitpunkt 12 Monate abschlossen (Tab. 1). Gründe für ein nicht abgeschlossenes Follow-up waren Wechsel der Patienten in die niedergelassenen Praxen oder ein Beobachtungsintervall kleiner 12 Monate. Die Therapieakzeptanz und -sicherheit waren insgesamt gut. Lebensbedrohliche unerwünschte Arzneimittelwirkungen (UAW) traten nicht auf. Es wurden im gesamten Beobachtungszeitraum keine sog. Rescue-Therapien mittels oraler Kortikosteroide oder Nebenhöhlenoperationen durchgeführt. Insgesamt gab es 6 Personen, die ihre Biologikatherapie im Laufe der Zeit wechselten, z. T. auch mehrfach. Gründe für Therapiewechsel waren Verträglichkeitsprobleme und fehlende Krankheitskontrolle. Die beobachteten UAW sind in Tab. 2 aufgeführt. Unter allen UAW kam es am häufigsten zu juckenden, teils rötlichen Veränderungen im Bereich der Einstichstellen. UAW traten unter Dupilumab-Therapie häufiger als mit anderen Präparaten auf. Es ist jedoch zu beachten, dass das bei Weitem am häufigsten eingesetzte Biologikum Dupilumab mit insgesamt 112 begonnenen Behandlungen war.

Das Patientenkollektiv zeigte zu Beginn der Therapie vornehmlich eine schwere, unkontrollierte Erkrankung: Sowohl in der VAS als auch im SNOT-22-Fragebogen erfüllten 62 % der Patienten diese Kriterien (Tab. 1). Im Durchschnitt wurden knapp über 3 Nasennebenhöhlenoperationen und bei allen Patienten ein Versuch einer ATAD bei den Patienten in der Vorgeschichte durchgeführt. Histologisch lag durchschnittlich eine mäßige bis starke Gewebseosinophilie im Nebenhöhlenpräparat vor, und auch im Blut zeigten sich teilweise stark erhöhte Konzentrationen von Eosinophilen als Hinweis auf das Vorliegen einer Typ-2-Entzündung.

### Veränderungen der klinischen Parameter

**Abb. 1:**
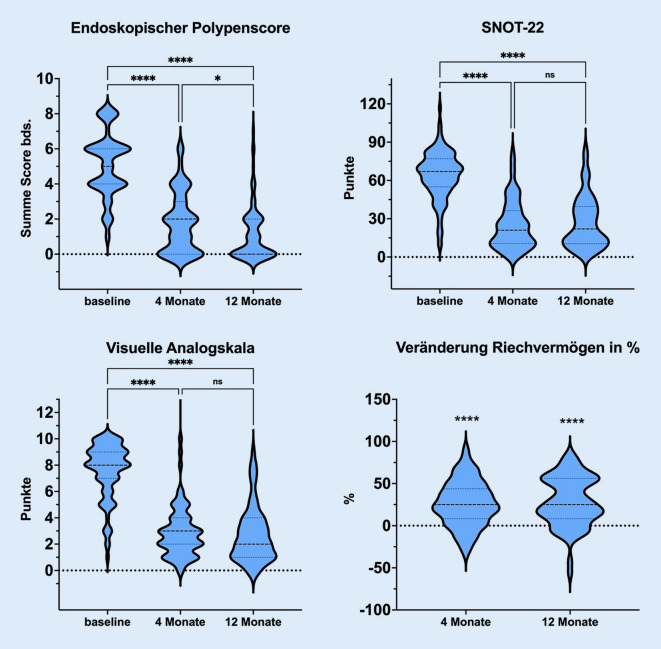
Durchschnittliche Absolutwerte (Violin-Graph) für endoskopischen Polypenscore, SNOT-22-Fragebogen, visuelle Analogskala und Veränderung des Riechvermögens. Visuelle Analogskala als Bewertung der subjektiven Gesamt-Krankheitslast von 0 bis 10. Riechvermögen als prozentuale Veränderung im Vergleich zur Baseline. *Asterisk* statistische Signifikanz mit *p* < 0,05; *ns *nicht signifikant, *SNOT* Sino-Nasal Outcome Test

**Abb. 2:**
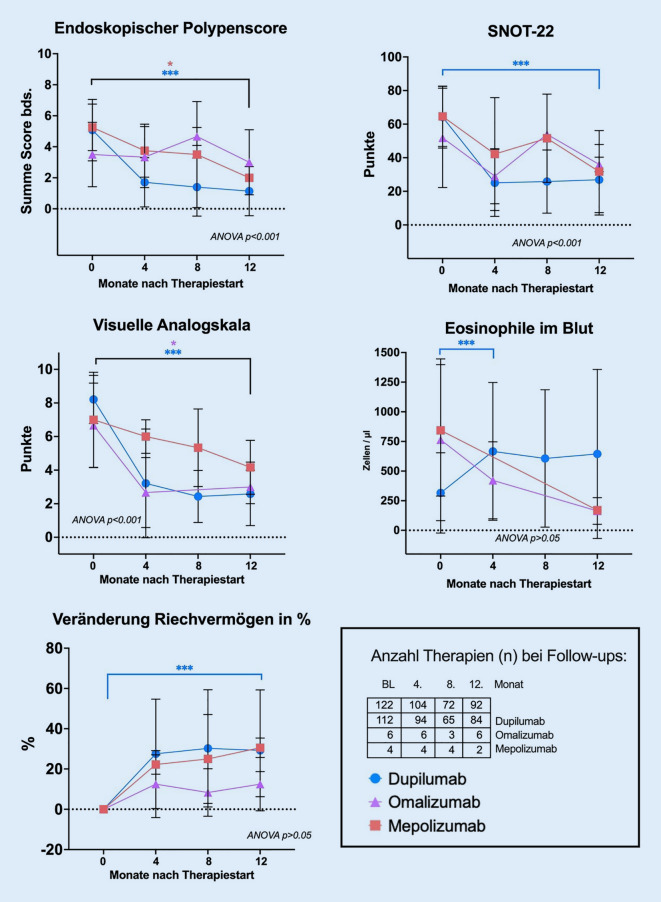
Durchschnittliche Entwicklung klinischer Parameter im Verlauf bis 12 Monate. *Asterisk* statistische Signifikanz gegenüber dem Ausgangswert mit *p* < 0,05;* BL *Baseline, *SNOT* Sino-Nasal Outcome Test, jeweilige *n* zum Zeitpunkt [x] rechts unten in der Tabelle angegeben. Für *n* [Präparat] s. Tab. 1. 2‑Wege-ANOVA mit statistischer Signifikanz über den zeitlichen Verlauf, nicht aber zwischen Präparategruppen

Die Add-on-Behandlung mit Biologika führte zu einer signifikanten Verbesserung der mit der VAS gemessenen subjektiven Krankheitslast von 7,6 auf 2,7 (*p* < 0,0001) und der mit dem SNOT-22 gemessenen Symptomlast/gesundheitsbezogenen Lebensqualität von 64,2 auf 27,7 (*p* > 0,0001; Tab. 1; Abb. 1). Die Behandlung mit jeweils einem der 3 Biologika Dupilumab, Mepolizumab und Omalizumab führte durchschnittlich zu einem verbesserten SNOT-22-Punktwert nach einem Jahr, wobei bei geringer Fallzahl für Mepolizumab und Omalizumab jeweils knapp keine Signifikanz erreicht wurde (Tab. 1; Abb. 2). Signifikant war auch die Verkleinerung der Gruppe der unkontrollierten, stark belasteten und subjektiv schwer erkrankten Patienten, gemessen am Gesamtkollektiv. So führte die Biologikatherapie zu einem Rückgang um 54,9 % auf lediglich 9 Fälle von stark belasteten und unkontrollierten Patienten (Tab. 1). Gleichzeitig nahm die Zahl der gut kontrollierten, leichtgradig erkrankten Fälle um 38,5 % zu auf *n* = 51 (*p* < 0,0001).

Die Polypenlast, gemessen anhand von Nasenendoskopie mit einem etablierten Score, konnte durch die Add-on-Therapie signifikant von 5,1 auf 1,1 nach einem Jahr der Therapie (Summationsscore beider Nasenseiten, jeweils 0 bis 4) gesenkt werden (Abb. 1). Zwischen dem ersten Follow-up nach 4 Monaten und dem 12-Monats-Follow-up zeigte sich ein zusätzlicher, signifikanter Rückgang der Polypenlast um rund 0,7 Punkte (*p* < 0,05; Abb. 1). Auch hier kam es zu einer Verringerung der Polypenlast in allen 3 Präparategruppen mit signifikantem Rückgang für Dupilumab und Mepolizumab im Follow-up nach einem Jahr (Tab. 1).

### Veränderung des Riechvermögens

Die subjektive Einschätzung der Verringerung des Geruchssinns lieferte vor Beginn der Biologikatherapie stark auffällige Werte. Im Durchschnitt wurde die Störung des Geruchssinns in der VAS bei 8,7 von maximal 10 Punkten gesehen. Wesentlich zuverlässiger ist die Messung des Riechvermögens mit standardisierten, psychophysikalischen Testmethoden [33]. Wegen der multizentrischen Natur der Studie wurden verschiedene Tests angewendet, deren Ergebnisse anhand der Berechnung von Quotienten und prozentualen Veränderung bestmöglich vergleichbar gemacht wurden. Der durchschnittliche Wert vor Therapie für den 16-Item-Sniffin’Sticks-Identifikations-Test lag bei 5,6 ± 4,3 Punkten, einer Anosmie entsprechend. Analog hierzu lag der Ausgangswert für die 12-Item-B-SIT und Sniffin’Sticks-Testung bei 3,6 ± 2,6 Punkten. Die Add-on-Therapie mit Biologika führte zu einer langanhaltenden Verbesserung der Testergebnisse zum Zeitpunkt 1 Jahr um durchschnittlich 28,7 % (*p* < 0,0001; Abb. 1). Trotz der kleinen Fallzahl für die Gruppe der Mepolizumab- und Omalizumab-Patienten ist auch hier ein klarer Trend der Verbesserung der Riechfunktion auch im Langzeitverlauf sichtbar (Abb. 2).

### Systemische Veränderungen

Die Messung von Immunglobulin der Gruppe E (IgE) sowie der eosinophilen Granulozyten im Blut hat sich in der Allergiesprechstunde zur besseren Einschätzung der Patienten mit CRSwNP etabliert [27]. Zwar ist die prognostische Aussagekraft und Korrelation mit klinischen Befunden limitiert, eignet sich aber dennoch für die Beurteilung der Aktivität der Typ-2-Inflammation in begrenztem Umfang. Unter Therapie mit Biologika kam es durchschnittlich zu einem kräftigen, signifikanten Anstieg der eosinophilen Granulozyten im Blut von rund 352 auf 607 Zellen/µl (Tab. 1). Am ehesten ist dieser erklärbar durch das Übergewicht von Dupilumab-Patienten im Kollektiv, da es unter jener Therapie häufig zu einem transienten Anstieg der Eosinophilen kommt. Unter Berücksichtigung der geringen Fallzahl ist dennoch auf den komplett gegenläufigen Verlauf der eosinophilen Granulozyten unter Therapie mit Mepolizumab und Omalizumab hinzuweisen (Abb. 2). Das Gesamt-IgE nahm unter Therapie mit Biologika signifikant von 263,6 U/ml auf 91,2 U/ml ab (*p* = 0,042; Tab. 1).

### Subjektive Therapiebewertung durch Patienten

**Abb. 3:**
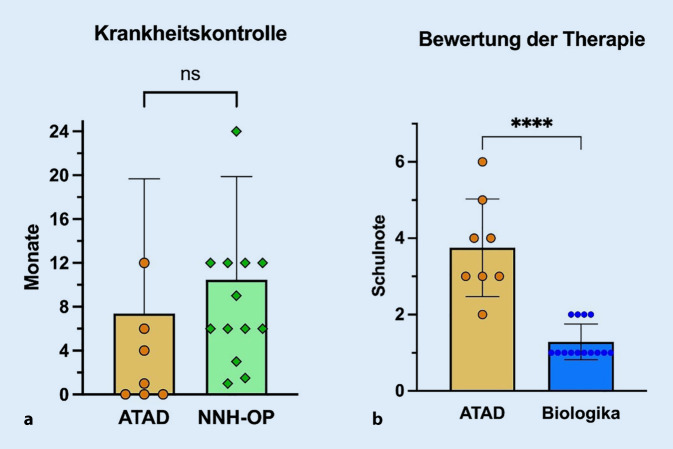
Einzel- und Durchschnittswerte für die Befragung zu Therapiekontrolle und Therapiebewertung. Anzahl *n* = 13, *Asterisk* statistische Signifikanz mit *p* < 0,05; *ASS* Acetylsalicylsäure,* ATAD *„aspirin-treatment after desensitization“, *NNH-Op.* Nasennebenhöhlenoperation, *ns* nicht signifikant. **a** Fragen 1 & 2. **b** Fragen 3 & 4. (Den Patienten wurden folgende 4 Fragen gestellt: 1) Wie viele Monate/Jahre haben sie nach der letzten Op. eine gute Krankheitskontrolle erlebt und wurde parallel ASS verabreicht?; 2) Wie viele Monate/Jahre haben Sie unter der ASS-Deaktivierung eine gute Kontrolle erlebt?; 3) Welche Gesamtnote [dt. Schulnote] würden Sie der ASS-Therapie mit/ohne Op. geben, unter Berücksichtigung von Verträglichkeit, Alltagstauglichkeit und Wirkung?; 4) Welche Gesamtnote [dt. Schulnote] würden Sie der Biologikatherapie geben, unter Berücksichtigung von Verträglichkeit, Alltagstauglichkeit und Wirkung?)

Die Therapieadhärenz hängt maßgeblich von Verträglichkeit, Zeitaufwand und subjektiv verspürtem Benefit ab. Bei der CRSwNP mit und ohne NERD handelt es sich um eine chronische Schleimhauterkrankung, sodass viele Therapiebausteine kontinuierlich von den Behandlern empfohlen werden. Die aktuell etablierten, medikamentösen Ansätze der CRSwNP-Behandlung (topisches Steroid, ATAD, Biologika) sind solche Dauertherapien, die alle in die Studie eingeschlossenen Patienten in der Vorgeschichte bereits angewendet hatten oder dies aktuell tun. Die Patienten wurden daher um eine persönliche Bewertung und Gegenüberstellung anhand von 4 Fragen gebeten (Abb. 3). Die Dauer der Krankheitskontrolle unter ATAD und nach einer Nasennebenhöhlenoperation ohne ATAD wurde von den Patienten als ähnlich empfunden (7,4 ± 12,3 Monate vs. 10,5 ± 9,4 Monate; *p* > 0,05; Abb. 3). Die Gesamtbewertung der medikamentösen Therapieformen ATAD und Biologika fiel mit fast −2,5 deutschen Schulnoten signifikant besser für die Biologika aus (Abb. 3). Dies unterstreicht die bislang beobachtete, gute Therapieakzeptanz der Add-on-Therapie mit Dupilumab, Mepolizumab oder Omalizumab.

## Diskussion

Die Add-on-Biologikatherapie der CRSwNP mit NERD stellt eine im längerfristigen Verlauf wirksame, sichere und gut akzeptierte Therapie dar. Bislang lagen keine multizentrischen Real-World-Daten über den Einsatz verschiedener Biologika bei dieser Erkrankung vor. Für die Indikationsstellung verwendete Kriterien nach deutscher und europäischer Literatur [4, 28] zeigten eine zuverlässige Auswahl der Patienten, was sich am guten Ansprechen und dem großen Anteil an nur noch leichtgradig, kontrolliert erkrankten Personen nach einem Behandlungsjahr spiegelt. Die Verlässlichkeit der EPOS- und EUFOREA-Kriterien (European Forum for Research and Education in Allergy and Airway Diseases) konnte kürzlich ebenfalls in der Real-World-Studie von van der Lans et al. unter Beweis gestellt werden [34].

Bei den Patienten in der vorliegenden Studie handelte es sich um Personen mit anamnestisch gesicherter Unverträglichkeit auf Salicylate oder durch Provokation nachgewiesener Intoleranz auf ASS (NERD, AERD, M. Samter/Widal). Alle Patienten hatten sich in der Vorgeschichte Nasennebenhöhlenoperationen und ATAD zur Kontrolle ihrer Erkrankung unterzogen, befanden sich aber in einem unkontrollierten Stadium schwerer CRSwNP. Die Add-on-Therapie mit Biologika führte zu einer signifikanten Reduktion der subjektiv mit visuellen Analogskalen ermittelten Krankheitsintensität von 7,6 auf 2,7 nach einem Jahr (Gesamtkollektiv), gemessen im Vergleich zum Ausgangsbefund. Die überwiegende Zahl der Patienten in dieser Studie erhielt Dupilumab (*n* = 112), und der signifikante Effekt unter jener Therapie ließ sich im 12-Monats-Verlauf stabil darstellen. Albrecht et al. zeigten in ihrer Real-World-Studie mit Dupilumab bei CRSwNP einen ähnlichen Rückgang der VAS-Werte von 6,36 auf 2,39 [35]. In einer Post-hoc-Analyse des Studienkollektivs der SINUS-24- und -52-Studien, die zur Zulassung von Dupilumab für die Indikation CRSwNP führten, zeigte sich unter NERD-Patienten ein Rückgang des VAS von 7,99 auf 4,39 nach einem halben Jahr [22]. Omalizumab zeigte in der hier vorliegenden Studie eine signifikante Reduktion der VAS nach einem Jahr, und in einer anderen Studie über einen Zeitraum von 9 Monaten eine signifikante Verbesserung der VAS bei 16 Patienten [19]. Analog zur VAS-Verbesserung zeigte sich in dieser Studie eine signifikante Verbesserung der gesundheitsbezogenen Lebensqualität, gemessen mit dem SNOT-22-Fragebogen, um durchschnittlich 37 Punkte (minimale klinisch relevante Veränderung: 8,9 P.) [32]. Alle 3 untersuchten Biologika-Patientengruppen erlebten eine klinisch relevante Verbesserung in den ersten 12 Monaten der Therapie, wobei der langfristige Effekt für Mepolizumab und Omalizumab aufgrund der kleinen Gruppengröße in dieser Studie nicht zuverlässig angegeben werden kann. Ebenfalls eine Post-hoc-Analyse der SYNAPSE-Zulassungsstudie für Mepolizumab zeigte, dass NERD-Patienten in Bezug auf VAS und SNOT-22 nicht signifikant anders als Nicht-NERD-Patienten abschneiden [26]. Die Daten der vorliegenden Studie sind mit den limitiert verfügbaren Real-World- und klinischen Studiendaten vergleichbar und legen nahe, dass die Add-on-Biologikatherapie die subjektiven Parameter zu Krankheitsintensität und Lebensqualität verbessert und der positive Effekt durch Dupilumab bei NERD-Patienten langfristig anhält.

Der endoskopisch erhobene Polypenbefund ist ein Kernkriterium der klinischen Praxis und der Verlaufskontrolle von CRSwNP-Patienten. In der Regel zeigt sich eine gute Korrelation zwischen Polypenlast und klinischen Symptomen und damit der Krankheitsaktivität. Daher hat sich auch die Reduktion von Polypen unter jeweilig untersuchter Therapieform als ein Hauptaugenmerk für die Beurteilung der Wirksamkeit etabliert. Die Add-on-Therapie mit Biologika war in den jeweiligen Zulassungsstudien für eine signifikante Reduktion der nasalen Polypen verantwortlich (Omalizumab: etwa −1 Punkt nach 24 Wochen, Dupilumab: etwa −1,9 Punkte nach 24 Wochen, Mepolizumab: etwa −1 Punkt nach 52 Wochen) [15–17]. In der Studie von van der Lans et al. erlebten über 88 % der mit Dupilumab behandelten Patienten eine Reduktion der nasalen Polypen von initial mehr als 5 um mehr als 2 Punkte nach rund einem Jahr Therapie [34], Albrecht et al. berichteten von einer Reduktion von durchschnittlich 5,3 auf 1,4 nach 12 Monaten. In der vorliegenden Studie beobachteten die Autoren sogar einen noch größeren Rückgang der Polypen im endoskopischen Polypenscore von durchschnittlich 5,1 auf 1,1 nach 12 Monaten, der v. a. von den Subgruppen der Dupilumab-Patienten und Mepolizumab-Patienten getragen wurde (*p* < 0,0001 bzw. *p* = 0,04). Der Rückgang der nasalen Polypen war unter Mepolizumab mit −3,2 Punkten deutlicher als in der Vergleichsliteratur, muss aber noch in größeren Fallzahlen von NERD Patienten überprüft werden.

Neben der Reduktion der Polypenlast zeigte sich auch eine deutliche Verbesserung des Riechvermögens bei den NERD-Patienten unter Biologika. Nicht immer lässt sich die Veränderung des Riechvermögens mit den Endoskopiebefunden in Einklang bringen. Die große Fläche der Riechrinne und die Relevanz der retronasalen Verteilung von Duftstoffen kann lokal umschriebenes Polypenwachstum umgehen, andererseits ist der häufig beschriebene Totalverlust des Geruchssinns bei moderatem oder kleinem Polypenwachstum nicht immer nachzuvollziehen [36]. Gerade der rasche Anstieg von olfaktorischer Funktion nach Beginn der Biologikatherapie deutet darauf hin, dass auch lokale, makroskopisch nicht sichtbare Entzündungsphänomene die Riechfunktion beeinträchtigen können und im Umkehrschluss durch Biologikagabe eine rasche Zunahme der Riechfunktion ohne Polypenrückgang möglich ist [37, 38]. Auch in dem Patientenkollektiv der Autoren kam es zu einer raschen Verbesserung der Riechfunktion in subjektiven (VAS; Tab. 1) und semiobjektiven Parametern nach 4 Monaten (*p* < 0,001). Kurzzeitige Verschlechterungen bei Einzelfällen sind durch akute Entzündungen oder auch saisonal-allergische Phänomene allerdings die Regel und sollten in der allergologisch-rhinologischen Sprechstunde erwartet werden. Mitunter waren sie auch der Grund für einen Teil der Therapiewechsel, was letzten Endes im Arzt-Patienten-Gespräch bewertet und gemeinsam entschieden werden muss [39].

Die ASS-Desaktivierung („aspirin therapy after desensitization“; ATAD) im Sinne einer Dauergabe von niedrig- bis hochdosierter Acetylsalicylsäure stellt eine medikamentöse Therapiealternative für Patienten mit NERD dar [13]. Sie wird i. d. R. knapp nach einer erfolgten Nasennebenhöhlenoperation begonnen und zeigte auch in Langzeitstudien verbesserte Krankheitsparameter und Symptomlast in mehreren Studien, sowohl das begleitende Asthma als auch die CRS betreffend [12, 40]. Dem gegenüber steht eine große Gruppe von abgebrochenen ATAD, was oft mit Nebenwirkungen begründet wird und v. a. bei hohen Dosierungen von ASS vorkommt: Teilweise reduzierte sich das Patientenkollektiv um knapp 50 % im Laufe der Studien [12, 41, 42]. Neben der Verträglichkeit ist die Krankheitskontrolle ein Faktor für die Akzeptanz der Therapie, die in der Studie von Bertlich et al. zwischen 1 und 17 Monaten rangierte [14]. In der Studie der Autoren gaben die Patienten ähnliche Werte aus Erinnerung an ihre in der Vorgeschichte stattgehabte ATAD an und bemerkten keine signifikante Verlängerung des Intervalls im Vergleich zu einer Nebenhöhlenoperation allein – soweit bei limitierter Gruppengröße aussagekräftig (Abb. 3). Es ist zudem zu beachten, dass in dieser Studie nur Personen mit begonnener Biologikatherapie ausgewertet wurden, die den Benefit der ATAD also für sich persönlich als nicht ausreichend bewertet haben und sich im Therapiegespräch aktiv für eine Biologikatherapie anstelle einer erneuten ATAD entschieden. Die Studienergebnisse unterstreichen eine sehr gute bis gute Therapieakzeptanz und Bewertung für die Add-on-Therapie mit Biologika.

UAW wurden bei den Patienten der Autoren in 22 Fällen (18 %) dokumentiert. Die Häufigkeit von der Medikation zuzuschreibenden UAW wird in anderen Phase-III- und Real-World-Studien mit 6–25 % in ähnlicher Häufigkeit beschrieben [15–17]. UAW, die zum Therapiewechsel führten, lagen in 4 Fällen vor (Tab. 2). Die UAW werden unter Biologikatherapie sehr unterschiedlich von den Betroffenen bewertet, sodass ein Arzt-Patienten-Gespräch über die Schwere des Phänomens, Prognose und Möglichkeiten zu Therapiealternativen und damit verbundenen Chancen unausweichlich ist. Die Mehrzahl der Patienten wünscht i. d. R. eine Fortsetzung der Therapie. Eine Therapiepause legten 2 Patientinnen wegen Kinderwunschbehandlung oder erfolgreicher Schwangerschaft ein. Die unter Dupilumab-Behandlung bekannte Beobachtung eines Anstiegs der eosinophilen Granulozyten im Blut wurde auch in diesem Kollektiv der NERD-Patienten gezeigt (*p* < 0,0001). Alle Fälle bis auf einen konnten jedoch unter engmaschiger Beobachtung und regelmäßiger Kontrolle des Differenzialblutbilds unter Therapie bleiben, weil sich klinisch keine Beschwerden des Allgemeinzustands, der Lunge oder in anderen Organen zeigten. Ein Fall unter den NERD-Patienten wurde aufgrund der Eosinophilie auf Mepolizumab umgestellt. Eine Hypereosinophilie zeigte sich bereits in 7 von 440 Fällen in den Zulassungs-Phase-III-Studien SINUS-24/52 unter Add-on Therapie mit Dupilumab, davon 4 symptomatische mit 3 EGPA-Diagnosen (eosinophile Granulomatose mit Polyangiitis) [15]. Auch in der Indikation Asthma bronchiale sind Einzelfälle mit Vaskulitis unter Dupilumab bekannt [43]. Eine weitere Differenzierung in NERD und Nicht-NERD lag hier nicht vor, und in der Arbeit von Mullol et al. werden jene Fälle nicht thematisiert [3]. Da keine klaren Grenzwerte definiert sind, beschränkt sich die aktuelle Empfehlung auf regelmäßige Kontrolle der eosinophilen Zellen und des klinischen Zustands der Patienten.

Die Add-on-Therapie mit den Biologika Dupilumab, Mepolizumab und Omalizumab hat zu einem Wandel in der klinischen Praxis weltweit geführt. Die historisch als schwer bis kaum kontrollierbaren Formen der CRSwNP, oft vergesellschaftet mit NERD, können gemäß aktuellen Leitlinien und Positionspapieren mit Biologika „in-label“ behandelt werden und so Rezidivoperationen und orale Steroidgaben vermieden werden [27, 28, 44]. Die bisherigen Real-World-Studien für Gesamtpopulationen und die hier vorgestellte Studie legen nahe, dass schwer erkrankte CRSwNP + NERD-Patienten mit ATAD und Operationen in der Vorgeschichte signifikant von der Add-on-Therapie mit Biologika über einen langen Zeitraum profitieren [34, 35, 45]. Der Biologikatherapie wird sogar eine Steigerung der Salicylat-Toleranz in einigen Studien zugeschrieben [23, 46], ein Nebeneffekt, der bisher aber unzureichend untersucht ist und den Patienten nicht in Aussicht gestellt werden sollte. Die bisherigen Subgruppenanalysen zu ATAD innerhalb des Kollektivs von CRSwNP unter Biologikatherapie stützten sich auf anamnestische Angaben bezüglich des Vorliegens eines NERD. Die vorliegende Studie umfasste jedoch Patienten mit ärztlich diagnostiziertem NERD und stattgehabter ATAD, was eine bessere Aussage auf den Behandlungsverlauf für NERD zulässt. Um jedoch den Stellenwert der ATAD und der Chirurgie in der Ära der Biologika in Zukunft besser zu ermitteln, bedarf es weiterer prospektiver Studien mit speziellen Subgruppen innerhalb der Population mit CRS [18]. Der ATAD wird ein günstigeres Kosten-Nutzen-Verhältnis zugeschrieben, wenngleich die geringere Verträglichkeit und Therapieakzeptanz dem entgegenzusetzen sind [25, 47]. Auch eine duale Therapie mit Biologika und ATAD im Fall unzureichenden Ansprechens auf manche Parameter – eine gute Verträglichkeit vorausgesetzt – könnte in Zukunft vermehrt eingesetzt werden, wenn ein gemischtes Ansprechen auf eines der beiden medikamentösen Konzepte vorliegt [48]. Dies wird aktuell insbesondere vor dem Hintergrund einer gewissen Toleranzinduktion gegenüber NSAID in der Wissenschaft diskutiert

Die retrospektive Natur der Daten und die stark asymmetrische Gruppengröße führen zu relevanten Limitationen der Aussagekraft dieser Studie. Auch die Verlaufsbeurteilung der unteren Atemwegserkrankung mittels routinemäßig durchgeführter Lungenfunktion und/oder Asthma Control Test bleibt ein Plan für zukünftige Forschungsvorhaben. Ein Direktvergleich zwischen Biologikapräparaten war nicht das Ziel des Vorhabens und sollte in zukünftigen Studien untersucht werden. Alle 3 hier untersuchten Präparate führten bei diesem multizentrischen Kollektiv von NERD-Patienten zu Symptomverbesserung und -kontrolle bei gutem Sicherheitsprofil. Im Einsatz verschiedener Biologika liegt jedoch auch eine Stärke der aktuellen Studie, denn es existieren nur sehr limitierte Daten zum Einsatz von Mepolizumab und Omalizumab außerhalb klinischer Studien. Der Rücklauf der Fragebögen oder telefonischen Kontakte war sehr limitiert. Die standardisierte Verlaufsbeobachtung von NERD-Patienten aus mehreren Zentren liegt nach Wissen der Autoren noch nicht vor und spiegelt die Realität aus der Klinik näher wider als Phase-III-Studien und monozentrische Erfahrungen. Die Zukunft der CRS-Therapie wird mit steigender Zahl von Biologikapatienten, -präparaten und verschieden gearteter Therapieversuche in der Vorgeschichte unübersichtlicher, weshalb die Hoffnung auf zukünftigen Registerstudien liegt, um mehr über die Wirksamkeit in kleinen Subgruppen wie der NERD zu erfahren.

## Fazit für die Praxis

- Die Add-on-Biologikatherapie der chronischen Rhinosinusitis mit Nasenpolypen (CRSwNP) mit Nichtsteroidale-Analgetika(NSAID)-Intoleranz („NSAID-exacerbated respiratory disease“, NERD) stellt eine im längerfristigen Verlauf wirksame, sichere und gut akzeptierte Therapie dar.
- Zukünftige Studien lassen eventuell mehr Aussagen über eine mögliche sequenzielle Abfolge von Operation, Acetylsalicylsäure(ASS)-Desensibilisierung (ATAD) und Biologikatherapie im Sinne neuer Therapiealgorithmen zu.
